# 442. Unveiling Factors Associated with Mortality in Mucormycosis Patients: Insights from Second and Third Waves of COVID-19 Pandemic in India

**DOI:** 10.1093/ofid/ofad500.512

**Published:** 2023-11-27

**Authors:** Vagisha Sharma, Kushal Kriplani, Isha Preet Tuli

**Affiliations:** Vardhman Mahavir Medical College and Safdarjung Hospital, New Delhi, Delhi, India; Vardhman Mahavir Medical College and Safdarjung Hospital, New Delhi, Delhi, India; Vardhman Mahavir Medical College and Safdarjung Hospital, New Delhi, Delhi, India

## Abstract

**Background:**

Mucormycosis is one of the most aggressive opportunistic fungal infections. Though conventionally limited to the demographics of immunocompromised and those on the diabetes spectrum. It has recently propelled to the frontline in investigations of COVID-19 complications. For patients present in varied stages and severity of Mucormycosis, it's crucial to discuss the presenting factors that impact mortality in order to provide more intensive care and treatment from the beginning to those more at risk of death.

**Methods:**

All patients presenting to a tertiary care hospital in New Delhi, India with clinicopathological confirmed Mucormycosis were enrolled in the study. Detailed history and physical examination were performed, and a questionnaire of patient demographic details and comorbidities was filled out.

**Results:**

There were 75 patients who met the inclusion criteria and were assessed after obtaining informed consent. Of these 75, 64 patients survived. The most common comorbidity in the study population was Diabetes spectrum(89.33%), followed by antecedent COVID-19 infection(64%) and hypertension(12%). Despite a high prevalence, none of these was significantly associated with mortality. The only comorbidity associated with mortality was Coronary Artery Disease(p-value< 0.05). The symptomatology at presentation was also assessed. Most commonly reported symptoms at presentation were Orbital/facial edema(45.33%), nasal discharge(29.33%), nasal block(29.33%) and diminution of vision(26.66%). The presence of proptosis at presentation in the patient was found to be significantly associated with a mortality outcome(p-value< .0.05). The glycemic control at presentation was also measured and compared between the survivors and non-survivors, however, no significant association was found.

Mucormycosis patient with Proptosis

**Figure:**
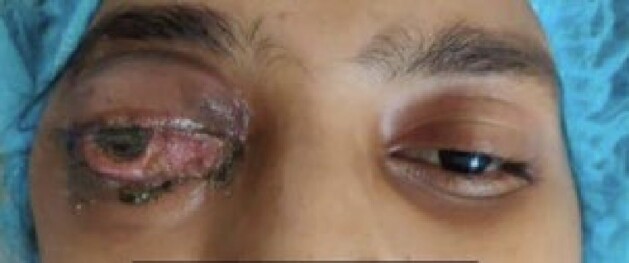

**Conclusion:**

Mucormycosis patients with presenting symptoms of proptosis should be prognosticated early and managed in the intensive care unit from the beginning due to the increased risk of mortality. Also, patients with coronary artery disease should be similarly given special attention and care due to the association with mortality. Other factors like antecedent COVID-19, diabetes, hypertension, tobacco and alcohol use and the extent of hyperglycemia do not impact patient outcome

**Disclosures:**

**All Authors**: No reported disclosures

